# Melusin gene (ITGB1BP2) nucleotide variations study in hypertensive and cardiopathic patients

**DOI:** 10.1186/1471-2350-10-140

**Published:** 2009-12-17

**Authors:** Valeria Palumbo, Ludovica Segat, Lara Padovan, Antonio Amoroso, Bruno Trimarco, Raffaele Izzo, Giuseppe Lembo, Vera Regitz–Zagrosek, Ralph Knoll, Mara Brancaccio, Guido Tarone, Sergio Crovella

**Affiliations:** 1 Servizio di Genetica, IRCCS Burlo Garofolo, via dell'Istria 65/1, Trieste 34100, Italy; 2 Laboratorio de Imunopatologia Keizo Azami (LIKA), Federal University of Pernambuco, Av Professor Moraes Rego, S/N - Cidade Universitária, CEP 50732-970, Recife (PE), Brazil; 3 Dipartimento di Genetica Biologia e Biochimica, Università di Torino, via Santena 19, Torino 10126, Italy; 4 Dipartimento di Medicina Clinica e Scienze Cardiovascolari e Immunologiche, Università degli Studi di Napoli Federico II, Via Sergio Pansini 5, Napoli 80131, Italy; 5 Dipartimento di Angiocardio Neurologia, IRCCS Neuromed, via Atinense 18, Pozzilli (IS) 86077, Italy; 6 Berlin Institute for Gender in Medicine (GiM) and Center for Cardiovascular Research (CCR), Charitè - University of Medicine Berlin, Hessische Str 3-4, Berlin 10115, Germany; 7 Cardiovascular Molecular Genetics, Heart Centre, Georg August University, Robert Koch Straße 40, Göttingen 37075, Germany; 8 Molecular Biotechnology Center, Università di Torino, via Nizza 53, Torino 10126, Italy

## Abstract

**Background:**

Melusin is a muscle specific signaling protein, required for compensatory hypertrophy response in pressure-overloaded heart. The role of Melusin in heart function has been established both by loss and gain of function experiments in murine models. With the aim of verifying the hypothesis of a potential role of the Melusin encoding gene, *ITGB1BP2*, in the modification of the clinical phenotype of human cardiomyopathies, we screened the *ITGB1BP2 *gene looking for genetic variations possibly associated to the pathological phenotype in three selected groups of patients affected by hypertension and dilated or hypertrophic cardiomyopathy

**Methods:**

We analyzed *ITGB1BP2 *by direct sequencing of the 11 coding exons and intron flanking sequences in 928 subjects, including 656 hypertensive or cardiopathic patients and 272 healthy individuals.

**Results:**

Only three nucleotide variations were found in patients of three distinct families: a C>T missense substitution at position 37 of exon 1 causing an amino acid change from His-13 to Tyr in the protein primary sequence, a duplication (IVS6+12_18dupTTTTGAG) near the 5'donor splice site of intron 6, and a silent 843C>T substitution in exon 11.

**Conclusions:**

The three variations of the *ITGB1BP2 *gene have been detected in families of patients affected either by hypertension or primary hypertrophic cardiomyopathy; however, a clear genotype/phenotype correlation was not evident. Preliminary functional results and bioinformatic analysis seem to exclude a role for IVS6+12_18dupTTTTGAG and 843C>T in affecting splicing mechanism.

Our analysis revealed an extremely low number of variations in the *ITGB1BP2 *gene in nearly 1000 hypertensive/cardiopathic and healthy individuals, thus suggesting a high degree of conservation of the melusin gene within the populations analyzed.

## Background

Melusin is a protein specifically expressed in heart and skeletal muscles where it binds to the cytoplasmic domain of β1 integrin, the membrane receptor anchoring the sarcomeres to the plasma membrane [1]. The role of Melusin in heart function has been established both by loss and gain of function experiments by generating a Melusin-null mouse lacking Melusin expression, and a Melusin transgenic mouse that over-expresses the protein in cardiomyocytes. The phenotype of these mice clearly indicates that Melusin is not required for heart development, sarcomere organization or cardiac function in basal conditions [2]. Melusin ablation, however, strongly impairs the left ventricle hypertrophy response to pressure overload, and dramatically accelerates the transition to cardiac dilation [2]. An opposite phenotype is observed when Melusin is over-expressed in the heart of transgenic mice. The left ventricles of these mice retain concentric compensatory hypertrophy with full contractile function and are protected from dilation when subjected to long-standing pressure overload [3]. These functional properties are accompanied by protection from cardiomyocyte apoptosis and lack of stromal tissue deposition, hallmarks of beneficial heart remodeling. Interestingly, endogenous Melusin levels are up-regulated during the initial phase of compensatory hypertrophy in mice subjected to aortic banding, but return to basal levels in heart that have undergone the transition toward dilation [3].

Moreover, the expression and regional distribution of Melusin in pressure-induced left-ventricular hypertrophy due to aortic stenosis (AS) was investigated in humans [4]: in normal hearts, Melusin was found in the myocytes with a uniform regional distribution, while Melusin staining, mRNA and protein were significantly decreased in AS hearts. The reduction in Melusin expression parallels the functional cardiac impairment in human AS.

At biochemical level, Melusin controls the phosphorylation of AKT and GSK3β in response to mechanical load. In fact, lack of Melusin leads to impaired phosphorylation of these proteins, while Melusin over-expression causes their over-phosphorylation in response to mechanical stimuli [2,3]. AKT is known to control phosphorylation of mTor, p70S6 and GSK3β, three serine/threonine kinases responsible for increased protein synthesis and cardiomyocyte hypertrophy [5]. Evidences from different laboratories [6,7] indicate that these molecules control increased cardiomyocyte size and concentric hypertrophy and trigger a beneficial compensatory cardiac hypertrophy.

Melusin is thus dispensable in physiological working conditions, but is required to trigger the beneficial hypertrophic response, and prevent left ventricle dilation in condition of exceptional mechanical overload. These properties qualify Melusin as a gene potentially affecting the evolution of the pathological status in cardiomyopathies.

With the aim of verifying the hypothesis of a potential role of the Melusin encoding gene, *ITGB1BP2*, in the modification of the clinical phenotype of human cardiomyopathies, we screened the entire coding region of *ITGB1BP2 *gene (Xq12-q13) and the intronic flanking regions looking for genetic variations possibly associated to the pathological phenotype in three selected groups of patients affected by hypertension and dilated (DCM) or hypertrophic (HCM) cardiomyopathy.

## Methods

### Patients

For this study we analyzed genomic DNA from the following patients and controls:

- 285 not related chronic hypertensive patients (average age at first admission 59 years) without cardiac hypertrophic remodeling, from Naples Hospital - Hypertension Diagnosis and Care Outpatient Clinic (HDCOC) of the Federico II University, Naples, Italy.

- 106 not related patients with primary hypertrophic cardiomyopathy (HCM) (average age at first admission 53 years) and 85 not related patients with primary dilated cardiomyopathy (DCM) (average age at first admission 50 years) from the German Heart Institute Berlin, Germany (DHZB).

- 41 not related patients with severe HCM or Hypertrophic obstructive cardiomyopathy (HOCM) and 139 not related patients with primary DCM from the Heart Centre Göttingen, Germany (HCG).

- 80 blood donors (average age 45 years, 30 from IRCCS Burlo Garofolo Blood Bank, Trieste, Italy and 50 from Naples Hospital Blood Bank, Italy) and 192 subjects (average age 50 years) from the DHZB with no history of DCM, HCM or other cardiovascular diseases, all not related were enrolled as healthy controls.

Additional information regarding patients' characteristics are reported on table 1.

**Table 1:** Patients' characteristics

	HDCOC (Naples)	DHZB (Berlin)	DHZB (Berlin)	HCG (Goettingen)	HCG (Goettingen)
Disease	chronic hypertensive patients without cardiac hypertrophic remodeling	primary hypertrophic cardiomyopathy (HCM)	**primary dilated cardiomyopathy (DCM) (no hypertension)**.	primary DCM	severe HCM or Hypertrophic obstructive cardiomyopathy (HOCM)
# of patients analyzed	285	106	85	139	40
Age (year)	60.3 ± 0.8	(at echo) 53 ± 1.35	(at echo) 50 ± 1.0	(at echo) 58.2 ± 14.0	(at echo) 56.8 ± 21.2
Sex M/F (%)	66.1/33.9	61/39	77.6/22.4	80/20	62.5/37.5
BMI Kg/m^2^	27.6 ± 0.3	27.3 ± 0.42	26.3 ± 0.41	28 ± 5.5	27.9 ± 5.9
LVEDD (mm)	50.9 ± 0.3	46.0 ± 0.73	69.9 ± 1.0	63.5 ± 8.1	53.2 ± 6.7
LVEF (%)	*n.a*.	*n.a*.	24.8 ± 0.66	30.2 ± 9.9	49.4 ± 12.9
IVS (mm)	10.9 ± 0.11	18.1 ± 0.47	*n.a*.	10.6 ± 1.8	14.6 ± 3.7
PW (mm)	9.4 ± 0.07	*n.a*.	*n.a*.	*n.a*.	*n.a*.
FS (%)	34.9 ± 0.3	33.7 ± 1.04	*n.a*.	*n.a*.	*n.a*.
SBP (mmHg)	160.7 ± 1.4	*n.a*.	*n.a*.	*n.a*.	*n.a*.
DBP (mmHg)	100.7 ± 1.0	*n.a*.	*n.a*.	*n.a*.	*n.a*.
Benign hypertension (%)	*n.a*.	37	*n.a*.	21.5	7.5
Diabetes melliyus (%)	6	7.6	*n.a*.	7.15	2.5
Hyperlipoproteinaemia (%)	37.7	32	*n.a*.	*n.a*.	*n.a*.
Smoking history (%)	26.2	47	*n.a*.	*n.a*.	*n.a*.
Positive family history (%)	82 (Hypertension)	45 (HCM)	18 (DCM)	20 (DCM)	27.5 (HCM or HOCM)

BMI: Body mass index. LVEDD: left ventricle end diastolic diameter. LVEF: left ventricle ejection fraction. IVS: inter ventricular septum. PW: posterior wall. FS: fractional shortening. SBP: sistolic blood pressure. DBP: diastolic blood pressure. *n.a*.: information not available.

The groups of HCM and DCM patients from DHZB (Berlin) have been examined also for sarcomeric gene mutations in previous studies. In particular 6,5% of DCM patients were found to carry mutations in beta-myosin heavy chain (*MYH7*) and myosin-binding protein C (*MYBPC3*) but not in troponin T (*TNNT2*), and alpha-tropomyosin (*TPM1*) [8]. In addition, 32% of HCM patients were found to carry mutations in myosin-binding protein C3 (*MYBPC3*), beta-myosin heavy chain (*MYH7)*, cardiac troponin T (*TNNT2*), cardiac troponin I (*TNNI3*) and alpha-tropomyosin (*TPM1*). No disease-causing mutation was found in cardiac troponin C (*TNNC1*) [9,10].

This project has been approved by the ethical committee of the Department of Genetics, Biology and Biochemistry of the University of Torino and informed consent was obtained from all the participants.

### *ITGB1BP2 *nucleotide variation scanning

Genomic DNA was extracted from peripheral whole blood with the GenomePrep extraction kit (Amersham-Pharmacia, Buckinghamshire, UK) following manufacturer's protocols.

PCR primers for *ITGB1BP2 *coding and flanking intron sequences amplification were designed by using the Primer Express 2.0 software (Applied Biosystems, Foster City CA) on the basis of the genomic human sequence (NM_012278; NC_000023). Primer sequences are reported in Table 2. Sequencing (both strands) was performed by using the Big Dye Terminator sequencing kit (Applied Biosystems, Foster City CA). Sequence reactions were run on an ABI 3130 Genetic Analyzer (Applied Biosystems) and the results handled with the Seqscape 1.0 software (Applied Biosystems).

**Table 2:** Primers used for PCR amplification of the 11 exons and intron flanking regions of the ITGB1BP2 gene

Exon	Exon size (bp)	Primer Forward	Primer Reverse	Amplicon size (bp)
**1**	63	TCAACCAACGCTTCCATG	GGCTGTAAGATCACTCAG	214
**2**	49	AATTGGTCTGAGTGATCTT	CAGATCAGCTTAGCTTCC	279
**3**	56	GGAAGCTAAGCTGATCTG	CTAGTATAGATAAGGAGA	168
**4**	145	ATTTATGTACTTGGATTC	CTAGTTCCCAGTCCTCAA	302
**5**	94	AACTAAATCACCACCCTT	ATTGCCACTTCCACTAAA	180
**6**	55	TGAGTGGGGGGATGGATA	AGGGCCTTGGTAAACCTA	227
**7**	70	CCCAGGATGTGATGCT	GTAGGAAGATAATTCAACTGAATT	304
**8**	99	GCTATGAGGCTATGAGAC	TGGCTATGTGGTACAGAG	273
**9**	113	CACGTGCATCATGAGAAC	CTCTGCCTGTGAGATTGT	254
**10**	62	CAACCTGTGGATCATACC	TCCAGTCTAGCCGCTCTT	222
**11**	227	CTCCTCCTCAGGTCATAA	TCAACTGTCTGGTGTCAC	256

### Minigene splicing assay

Genomic DNA from patients with IVS6+12_18dupTTTTGAG and 843C>T mutations and from a wild-type control were amplified with appropriate primers containing EcoRI and XhoI 5' site in order to generate fragments that contain Melusin gene sequence spanning from exon 5 to 7 (carrying the IVS6+12_19dup18dupTTTTGAG mutation, primer Fw 5'-CCGGAATTCATGAAGTCAGAGTTGCCT-3', primer Rev 5'-CCGCTCGAGCCCCTCATGGAAT-3') and Melusin gene sequence spanning from exon 9 to 11 (carrying the 843C>T polymorphism, primer Fw 5'-CGGAATTCCGATGCTCCCA-3', primer Rev 5'-TGCACTCGAGTCACATTCTTTCAACT-3'). These fragments, following Klenow kinasation, were cloned into Sma-digested pUC plasmid for screening and sequencing. The fragments presenting the correct sequence, obtained from these constructs, were subcloned EcoRI/XhoI into the polylinker site of the pCDNA3 vector. HeLa cells have been transfected with the minigene constructs using the Effectene Transfection Kit (Qiagen). RNA extraction has been performed after 24 h following standard procedures, and reverse transcription-polymerase chain reaction (RT-PCR) has been done with primers pairing with SP6 and T7 regions in the plasmid for the Melusin exon 5-7 minigene, and with specific oligonucleotides pairing with the beginning of the exon 9 (primer Fw 5'-AGCATCTTGCCGCCATGATTGGC-3') and an 11 exon internal region, close to the stop signal (primer Rev 5'-TAGACATCAACTGTCTGGTG-3') for the Melusin exon 9-11 minigene. RT-PCR products have been analyzed on a 2% agarose gel and amplicon sequences verified by direct sequencing.

## Results

A total of 928 subjects including 656 patients from three different groups and 272 controls were screened.

Only three nucleotide variations, two in hypertensive patients of the HDCOC cohort and one in the HCM patients group from the DHZB group were evidenced. (Table 3).

**Table 3:** ITGB1BP2 nucleotide variations identified in this study

Patient/sex and origin	Diagnosis	Mutation	Mutation status	Mutation details
#1/M HDCOC*	Hypertensive individuals with eccentric left ventricle remodeling	843 C>T Silent mutation	Hemizygosis	In exon 11, 8 bp downstream an exonic splicing enhancer - ESE - consensus sequence
#2/F HDCOC*	Hypertensive individuals with eccentric left ventricle remodeling	IVS6+12_18dupTTTTGAG intronic	Heterozygosis	In intron 6 near the 5'donor splice site (10 bp downstream)
#3/F DHZB^$^	Hypertrophic cardiomyopathy (HCM)	37 C>T Missense mutation	Heterozygosis	In exon 1, causes a His13Tyr substitution in the CHORD domain

* HDCOC: Hypertension Diagnosis and Care Outpatient Clinic of the Federico II University, Naples, Italy.

^$^DHZB: German Heart Institute, Berlin, Germany.

M = male

F = female

The first variation is a C>T transition at nucleotide 843 in the 5' region of exon 11 in close proximity to the preceding intron. This is a silent substitution, which does not modify the coded amino acid. The 843C>T single nucleotide polymorphism (SNP) was found in hemizygosis in a male patient (#1) from HDCOC group characterized by hypertension, eccentric cardiac remodeling and who suffered from an ictus at the age of 66.

The second variation detected is a duplication, IVS6+12_18dupTTTTGAG, occurring in intron 6 near the 5'donor splice site and found in heterozygosis in a female patient (#2) from HDCOC group.

Both the 843C>T and IVS6+12_18dupTTTTGAG variations have not been found in healthy controls.

The third variation consists of C>T transition found in heterozygosis at position 37 of exon 1 in one HCM female patient (#3) from the DHZB group. The nucleotide exchange leads to a substitution of a conserved amino acid residue: histidine-13 to tyrosine. This patient developed HCM at age 38. This variation was not found in the 192 healthy controls from Germany as well as in the 80 Italian controls.

These were the only three nucleotide variations we observed in the whole coding region and flanking sequences of the *ITGB1BP2 *gene in the 930 individuals investigated (658 patients and 272 controls).

**Figure 1:**
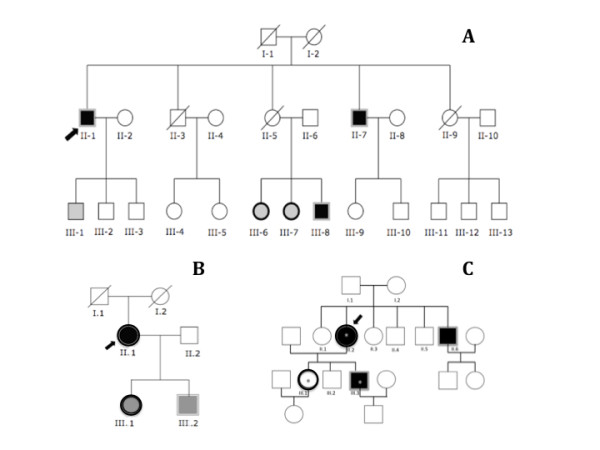
**A: pedigree of patient's #1 family**. Patient's #1, (II.1, the proband, indicated by the arrow) carries the 843C>T SNP in exon 11. Subjects II.1, II.7, III.8 are hemizygous (single border grey line) for the mutated allele while subjects III.6 and III.7 are heterozygous (single border black line). Black filled squares and circles: subjects with eccentric remodeling of the left ventricle; grey filled squares and circles: individuals with normal echocardiogram parameters. Information concerning clinical status, cardiac phenotype and *ITGB1BP2 *mutations are lacking in other subjects (empty circles and squares). **B**: pedigree of patient's #2 family. Patient's #2 (II.1, the proband, indicated by the arrow) carries the duplication IVS6+12_18dupTTTTGAG in intron 6. Subjects II.1 and III.1 are heterozygous (single border black line) for the mutated allele while subject III.2 is hemizygous (single border grey line). Black filled circle: subject with eccentric remodeling of the left ventricle; grey filled squares and circles: individuals with normal echocardiogram parameters. Information concerning clinical status, cardiac phenotype and *ITGB1BP2 *mutations are lacking in other subjects (empty circles and squares). **C**: pedigree of patient's #3 family. Patient's #3 (II.2, the proband, indicated by the arrow) carries the C>T missense mutation at position 37 in exon 1. Subjects II.2 and III.1 are heterozygous (single border black line) for the mutated allele while II.6 and III.3 are hemizygous (single border grey line). Black filled squares and circles: subjects characterized by HCM. Empty circles and squares: not affected subjects. Some members of this family also carry a mutation in the 3'UTR of the troponin T gene (asterisk).

We then constructed the family pedigree (Figure 1) of the three patients for which we found a mutation in the *ITGB1BP2 *gene.

For patient #1 (proband II-1, Figure 1A) we were able to identify 25 individuals through three generations. Unfortunately, clinical information and cardiac phenotype were lacking in 19 subjects. Three male patients (II-1 the proband, II-7 and III-8), all characterized by eccentric remodeling of the left ventricle, were hemizygous for the 843C>T SNP. Two females (III-6, III-7) with normal echocardiogram parameters were heterozygous for the mutation.

The family pedigree of patient #2 (proband II.1) is reported in Figure 1B. We encountered only six individuals through three generations. Proband II.1 is heterozygous for the IVS6+12_18dupTTTTGAG and is characterized by eccentric remodeling of the left ventricle. Two other members (one female III.1 and one male III.2) of the family carrying the IVS6+12_18dupTTTTGAG (III.1 heterozygous; III.2 hemizygous) are characterized by a normal echocardiogram.

**Figure 2:**
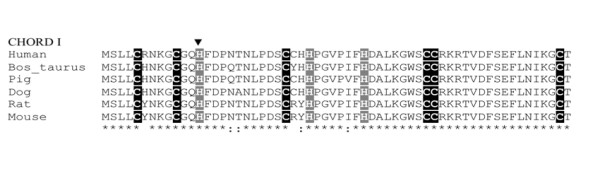
**Alignment of the primary sequence of melusin CHORD 1 domain in a number of mammalian species indicates the high degree of conservation of the gene**. The characteristic C (cysteine) and H (histidine) signature are highlighted. The position of the missense mutation detected in HCM patient causing His-13 > Tyr amino acid change is indicated by the arrowhead. (*) conserved residues (:) conserved substitutions.

The family pedigree of the HCM patient #3 (proband II.2) is depicted in Figure 1C. The C>T transition at position 37 in the exon 1, leading to an alteration of a conserved amino acid (histidin-13/tyrosine) in the CHORD 1 domain of the *ITGB1BP2 *gene (see Figure 2), was detected in the proband II.2 (heterozygous), in her brother II.6 (hemizygous), her daughter III.1 (heterozygous) and her son III.3 (hemizygous). Subjects II.2, II.6 and III.3 are affected by HCM.

The proband II.2 and her two sons III.1 and III.3 were known from previous analyses to carry a mutation in the 3'UTR of the troponin T gene (+6C/T 3'UTR). The troponin mutation was not present in the proband's husband and healthy son III.2, as well as in the proband's brother II.6 that was also characterized by HCM and the C>T transition at position 37 in the exon 1.

**Figure 3:**
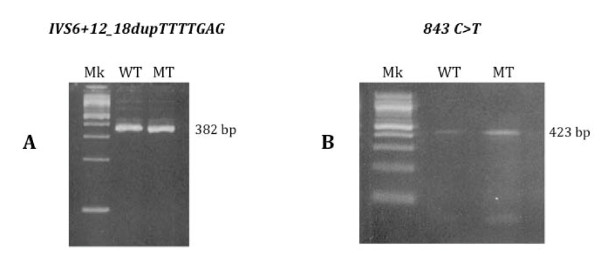
**A: Analysis of the splicing pattern by RT-PCR of the wild-type and IVS6+12_18dupTTTTGAG constructs after transfection in the HeLa cell line: both constructs reproduce the same splicing pattern (amplicon length 382 bp)**. Mk: 100 bp molecular ladder; WT: wild-type sample; MT: IVS6+12_18dupTTTTGAG sample. B: Analysis of the splicing pattern by RT-PCR of the wild-type and 843C>T SNP constructs after transfection in the HeLa cell line, showing that both constructs reproduce the same splicing pattern (amplicon length 432 bp). Mk: 100 bp molecular ladder; WT: wild-type sample; MT: 843C>T sample.

We then performed an *in-silico *analysis for both the 843C>T and IVS6+12_18dupTTTTGAG, with the aim of testing the possible effects of the nucleotide variation on exon splicing using the following softwares NNsplice http://www.fruitfly.org/seq_tools/splice.html, Cryp-Skip http://www.dbass.org.uk/cryp-skip/ and NetGene2 http://www.cbs.dtu.dk/services/NetGene2/: the results obtained indicated that the mutated sequences didn't introduce or delete any donor or acceptor splice site. In order to test the effect of the mutations *in-vitro*, we also performed a minigene splicing assay for both 843C>T and IVS6+12_18dupTTTTGAG, showing that the transcripts obtained from the cells transfected with the wild-type sequence and the mutated one, were the same both in terms of length (Figure 3) and of sequence of transcripts.

The missense substitution C>T at position 37 resulting in a H13Y substitution falls in a highly conserved region of Melusin corresponding to the CHORD 1 domain characterized by a unique Cys and His signature (Figure 2). To investigate the putative functional consequences of this mutation we performed a bioinformatics analysis by using the PolyPhen software http://genetics.bwh.harvard.edu/pph/index.html. The results of the analysis (PSIC score difference = 1.530) indicate that the H13Y substitution is predicted to be possibly damaging the protein structure/function.

## Discussion

The results of different studies [2,3,11], suggested a role for Melusin in cardiomyopathies as modifier gene, whose inactivation worsens the pathology and/or increases susceptibility to heart failure in response to different pathological conditions.

Analysis of the available data in the human genome database http://www.ncbi.nlm.nih.gov/Genbank/ indicated that Melusin *ITGB1BP2 *gene is highly conserved and only a limited number of single nucleotide polymorphisms have been described. In particular 35 SNPs are currently in the NCBI SNPs database http://www.ncbi.nlm.nih.gov/SNP/: 4 non-synonymous, 1 synonymous, 11 intronic, 19 in gene region. None of these SNPs, however, has been correlated with human pathologies and their frequency in the population has not been reported.

In this study we evidenced an extremely low number of variations in the *ITGB1BP2 *gene in nearly 1000 hypertensive/cardiopathic and healthy individuals, thus suggesting a high degree of conservation of the melusin gene, within the populations analyzed. In fact, only three genetic variants were detected in a total of 656 patients and 272 healthy controls screened by direct sequencing analysis; moreover the three variations have been found in three different patient and not in any control.

The 843C>T transition was found in a patient (#1) from HDCOC group of hypertensive patients that developed eccentric remodeling of the left ventricle. When analyzing the patient's family, three male subjects, all characterized by eccentric remodeling of the left ventricle, were hemizygous for the 843C>T SNP while the remaining three individuals with standard echocardiography profile showed the wild type DNA sequence. This SNP falls in the exon 11 coding region but is a silent substitution not affecting the coded amino acid. The sequence surrounding the 843C>T SNP, however, falls 3' to a consensus motif for an exonic splicing enhancer sequence (ESE), short nucleotide sequences that can stimulate splicing and regulate alternative splicing [12]. ESE sequences are present in most, if not all, exons, including constitutive ones and are thought to serve as binding sites for specific serine/arginine-rich (SR) proteins that promote exon definition by recruiting the splicing machinery. Nonsense, missense and even translationally silent mutations occurring in such ESE sequences can inactivate genes by inducing the splicing machinery to skip the mutant exons. Similarly, coding-region single-nucleotide polymorphisms might cause phenotypic variability by influencing splicing accuracy or efficiency [12,13]. This hypothesis has been verified by a functional assay: we performed an analysis of the 843C>T SNP, by using a minigene system in order to test if the 843C>T SNP may affects Melusin gene splicing; our results indicate that the nucleotide substitution seems not to affect the splicing, similarly to what suggested by an *in silico *analysis we performed. The length and sequence of the mutated transcript were the same of the wild-type ones. However, these results should be cautiously considered, since they are just an *in vitro *model that may not completely reflect what happens *in vivo *in the patients, and the Hela cell line model (available to us) is quite different from the cardiomyocytes. Taking in account these limitations, we can not exclude the possibility that, in the in vivo context of muscle cell, the mutation detected can alter the splicing introducing stop codon(s) which can ultimately lead to nonsense-mediated decay of the aberrant transcript, as already reported for mutations in other genes [14,15]. Testing this hypothesis requires analysis of Melusin transcripts in samples of skeletal muscle biopsies from patients, which, unfortunately, were not available for our study.

The second mutation detected in a hypertensive patient (#2) of the HDCOC group was the duplication IVS6+12_18dupTTTTGAG, occurring near the 5'donor splice site of intron 6. Such duplication was found in heterozygosis in a female patient presenting a modest cardiac remodeling. However, it was also found in an unaffected male as well as in an unaffected female, so this mutation reasonably does not correlate with the eccentric remodeling of the left ventricle and it is likely to represent an additional polymorphism of the Melusin gene whose association with the cardiac pathology is unlikely.

Similarly to what done for the 843C>T, also for the IVS6+12_18dupTTTTGAG we performed an *in silico *and a minigene splicing assay, without evidencing any difference in the transcript between the wild-type and the mutated sequence. However, the same arguments discussed for the 843C>T SNP, can be applied also to this mutation.

The third mutation detected is a C>T transition at position 37 in the exon 1 of the *ITGB1BP2 *gene and represents a missense mutation causing an amino acid change from His-13 to Tyr in the protein primary sequence. Such mutation falls in a highly conserved region of the Melusin molecule corresponding to the first CHORD domain characterized by a unique Cys and His signature [2] (Figure 2). The tertiary structure of Melusin CHORD domains has not been solved yet, and this fact restrains the possibility to predict the effect of the mutation. However, the substitution of an His residue, which is highly conserved and is part of the signature of the mammalian melusin CHORD 1 domain (Figure 2), strongly suggest that such mutation can modify the structure/function of the protein. This hypothesis, moreover, is also supported by preliminary bioinformatic analysis. The His/Tyr mutation in exon 1 was detected in heterozygosity in female patients II.2 and III.1 and in hemizygosity in male patients II.6 and III.3. No biological samples other than DNA were available from the subjects, thus impeding the possibility to perform functional study on the protein. The effect of this mutation remains to be disclosed, and it should also be noticed that while three of the patients (II.2, II.6, III.3) that presented the mutation are affected by HCM, one of the two heterozygote females (III.1) is not. This family also carries (II.2, III.1, III.3) an additional mutation in the 3' UTR of troponin T gene, whose molecular impact on the HCM status is unknown as well.

## Conclusions

In the present study we evidenced an extremely low number of variations in the *ITGB1BP2 *gene in nearly 1000 hypertensive/cardiopathic and healthy individuals, thus suggesting a high degree of conservation of the melusin gene, at least within the populations analyzed: only three genetic variants were detected in a total of 930 individuals. This very low frequency of polymorphisms is surprising in a gene whose ablation in an animal model system does not cause phenotypic alterations in basal conditions [2]. In fact, such strong sequence conservation is usually typical of genes whose alteration causes vital defects in embryos.

Nonetheless, in spite of a great conservation of the *ITGB1BP2 *gene, we have identified three new polymorphisms in three pedigrees that behave as private SNPs whose impact on the cardiovascular pathologies remains to be defined. Notwithstanding the fact that a preliminary functional assay on the role of two of these variations showed no effect, given the limitation of this approach, we believe that the impact of the 843C>T substitution in particular should deserve further functional investigations, since it has been evidenced in hemizygosis only in the affected members of a family with hypertension and eccentric left ventricle remodeling. It remains opened the question whether this variation should be considered as "private" variation specific of the family where it was associated with the clinical phenotype or if other subjects affected by CMD not considered in the present study could also present this variation. The last hypothesis is that 843C>T SNP could be simply a "private" marker that associated with the clinical phenotype in the family considered.

## Abbreviations

(AS): aortic stenosis; (DCM): dilated cardiomyopathy; (HCM): hypertrophic cardiomyopathy; (HOCM): hypertrophic obstructive cardiomyopathy; (HDCOC): Hypertension Diagnosis and Care Outpatient Clinic; (DHZB): German Heart Institute Berlin, Germany; (HCG): Heart Centre Göttingen, Germany; (LVEF): left ventricular ejection fraction; (LVEDD): left ventricular end diastolic diameter; (IVS): inter ventricular septum; (FS): systolic fraction; (RT-PCR): reverse transcription-polymerase chain reaction; (SNP): single-nucleotide polymorphism; (ESE): exonic splicing enhancer sequence.

## Competing interests

The authors declare that they have no competing interests.

## Authors' contributions

LS redacted the manuscript and performed the bioinformatic analysis. VP performed the DNA sequencing and minigene experiment. LP performed the DNA sequencing. AA conceived the study design. BT and RI performed the patients' clinical profile. GL performed the patients clinical profiling and study design. VRZ performed manuscript redaction and DNA sequencing. RK performed DNA sequencing and genotype phenotype correlation. MB performed genotype phenotype correlation. GT and SC conceived the study design and redacted the manuscript. All authors read and approved the final manuscript

## Pre-publication history

The pre-publication history for this paper can be accessed here:

http://www.biomedcentral.com/1471-2350/10/140/prepub
